# Camouflage efficiency in a colour‐polymorphic predator is dependent on environmental variation and snow presence in the wild

**DOI:** 10.1002/ece3.10824

**Published:** 2023-12-09

**Authors:** Charlotte Perrault, Chiara Morosinotto, Jon E. Brommer, Patrik Karell

**Affiliations:** ^1^ Department of Biology University of Turku Turku Finland; ^2^ Department of Bioeconomy Novia University of Applied Sciences Tammisaari Finland; ^3^ Department of Biology, Evolutionary Ecology Unit Lund University Lund Sweden; ^4^ Department of Ecology and Genetics University of Uppsala Uppsala Sweden; ^5^ Present address: Department of Biology University of Padova Padova Italy; ^6^ Present address: National Biodiversity Future Center (NBFC) Palermo Italy

**Keywords:** camouflage, climate change, colour, environmental, polymorphic, predator

## Abstract

Colour polymorphism can be maintained by colour morph‐specific benefits across environmental conditions. Currently, the amount and the duration of snow cover during winter decrease especially in northern latitudes, which can alter the potential for camouflage of animals with light and dark morphs. Tawny owls, *Strix aluco*, are colour‐polymorphic avian predators with dark (brown) and light (grey) colour morphs, where the grey morph is presumed to enjoy camouflage benefits under snowy conditions. We studied the camouflage potential of morphs in two tawny owls potential using passerines' probability to mob in the wild during winter with and without snow. For comparison with other seasons, we also repeated the experiment during spring and autumn. We found that grey tawny owls have a lower probability of being mobbed than the brown tawny owls only during snowy winters. The two colour morphs therefore experience differential benefits across snow conditions, which may help to maintain colour morphs in the population, although further warming of winter climate will reduce the potential for camouflage for grey tawny owls in northern latitudes.

## Graphical abstract

You better be a grey tawny owl, if you live in snowy conditions and want to minimize harassment. Using an experimental approach with stuffed tawny owls of the grey and brown morph, we show that grey tawny owls are less mobbed than brown tawny owls during snowy winters. In the other seasons, both morphs are equally detected.

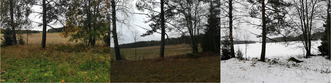

## 1 INTRODUCTION

Maintenance of phenotypic variation is puzzling given that natural selection is expected to erode it (Bulmer, 1985; Fisher, 1958; Hill & Mackay, 2004). Disruptive and frequency‐dependent selection as well as co‐evolutionary processes, among others, can maintain variation of selectively important traits (Ayala & Campbell, 1974; Bond & Kamil, 1998; Gigord et al., 2001; Hori, 1993; Mather, 1955). Animals can, for example, be polymorphic in terms of their colouration. Colouration is often heritable and colour morphs may vary in their fitness depending on the environment. For a predator, a colouration matching its background environment (i.e. camouflage) makes it less visible to its prey, thereby increasing its hunting success. For example, different colour morphs in the barn owl, *Tyto alba*, that vary from white to reddish, exhibit differential hunting success depending on moonlight conditions (San‐Jose et al., 2019).

Colour polymorphism is phylogenetically common among owl species (Strigiformes), where one‐third of all described species are polymorphic in colour (Galeotti & Sacchi, 2003). The tawny owl, *Strix aluco*, displays two genetically determined colour morphs: the grey morph and the brown morph. These morphs are determined by the irrevocable deposition of pheomelanin pigmentation in the plumage feathers during feather growth forming either a dark (brown) or a light (grey) phenotype (Brommer et al., 2005). To human observers being shown pictures, the grey tawny owl morph is more cryptic in snowy landscapes than the brown morph (Koskenpato et al., 2020). However, it is unclear if prey species, such as small passerines, perceive these two tawny owl morphs differently under natural conditions.

Prey have evolved antipredator behaviours to avoid direct, potentially lethal, predator encounters (Caro, 2005). In passerines, which are commonly preyed upon by tawny owls, this behaviour starts with the detection of the predator and signalling its presence to peers. Signals by a bird individual in the presence of a predator can either warn others or deter the predator from attack (Klump & Shalter, 1984). Then, other birds can join the first bird to form a group to harass the predator in a cooperative way, which is called mobbing. Mobbing motivates the predator to leave the area (Curio, 1978; Desrochers et al., 2002; Dominey, 1983; Flasskamp, 1994; Hurd, 1996; Pavey & Smyth, 1998; Pettifor, 1990; Wilson, 2000) and is hence likely to be energetically costly, thereby imposing a cost on predators for being detected.

Here, we quantify the probability a tawny owl is detected by small passerines and the probability these passerines form a mob. We first focus on the winter season where we expect the grey tawny owl morph to be more difficult to detect only when there is snow in the environment, since it is less conspicuous with this background (Koskenpato et al., 2020). Indeed, climate change is known to alter individuals' camouflage ability of animals adapted to snowy winters by reducing snow amount in the environment (Zimova et al., 2016). Second, we repeat the experiment in spring and autumn when there is more vegetation. Based on the findings of Koskenpato et al. (2020), we expect the brown morph to enjoy cryptic advantages relative to the grey morph in such environments.

## 2 MATERIALS AND METHODS

The experiments were conducted in the village Vassböle in South‐western Finland (central point (Labbas): 60.126004° N 24.049494° E). Fourteen locations were selected within the area, separated from each other by an average of 380.73 m (±172.02 m). A black feeder for bird seeds, a feeder for fat balls and a pole were installed at each location. Fat balls were provided during the winter times in order to attract more diverse communities of birds, this feeding resource is very attractive especially during cold periods. For repeatability purposes, we also provided fat balls in all other seasons. The feeders were hanging from a tree, and a pole was pressed into the ground 10 (±2) m from them. Each feeder was filled every week throughout all fieldwork seasons to attract the birds to the observation locations.

**FIGURE 1:**
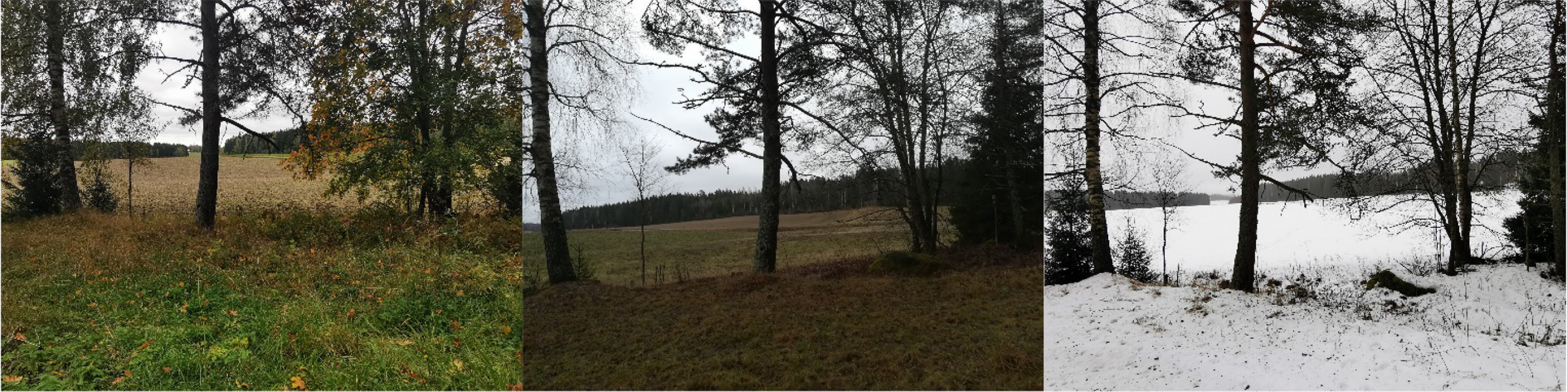
Pictures of one location from left to right in autumn, winter without snow and winter with snow.

The experiments were conducted four times. Once during winter without snow cover (December 2019 to March 2020), once during spring (May 2021), once during autumn (September to October 2021) and once during winter with snow cover (January to February 2022) (Figure 1).

Every observation was made between 7:00 and 14:00 when the birds are most active. The experiments started in December 2019 with one observer. Between February and March, two observers were present. After March 2020, only one of the two observers was performing the observations alone.

**FIGURE 2:**
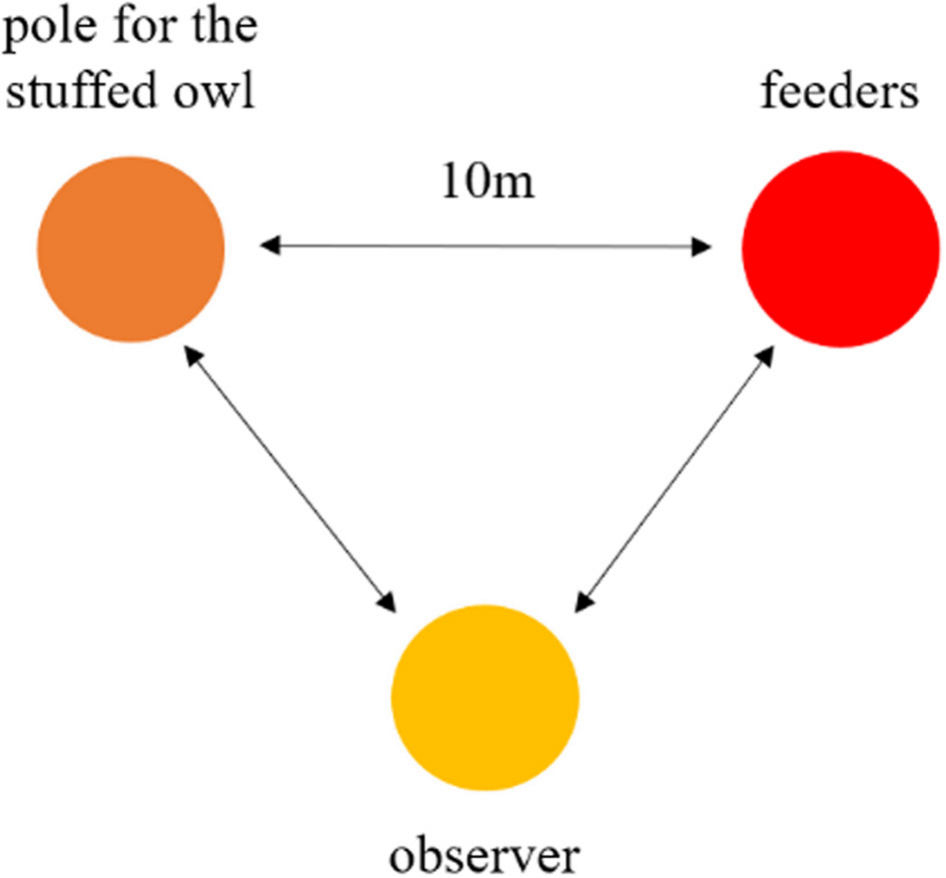
Scheme of the experimental design, seen from above.

Four stuffed tawny owls (*Strix aluco*, two grey and two brown morphs) were used and attached to a wooden pole. For each location five observations were conducted, one observation per each of the four stuffed owls (in random order) and a control (no stuffed owl). Arriving at one location, the observer(s) attached the stuffed owl pole with a rope to the pole already in place and took position equidistantly from the stuffed owl and the feeder to form a triangle from above (Figure 2).

After 1 min, the observer took the hood off from the stuffed owl. When the observer was back to the observation spot, the observation officially started. During control, the same process, without the stuffed owl, was conducted to minimize the differences between the control and experimental trials. The aim was to have each morph tested 28 times in each season (14 locations * 2 stuffed owls). Due to technical issues, the realized sample size varied a bit (Table 1). In addition to the 224 trails with a stuffed owl (Table 1), there were 56 controls (14 locations * 4 seasons) and hence 280 trials conducted in total.

**TABLE 1:** Table describing the number of trial per each stuffed owl morph per season.

	Brown stuffed owl	Grey stuffed owl	Total
Spring	25	27	52
Autumn	27	28	55
Winter without snow	29	28	57
Winter with snow	30	30	60
Total	111	113	224

During each trial, the behaviour of the mobbing birds was monitored. Behaviours observed were: the time until the first bird approaches the stuffed owl, its species, the time until the mob occurs and the distance of the approaching and mobbing birds from the stuffed owl. Several behavioural traits were considered and defined as:

- a *detection*, when a bird came closer to the stuffed owl, looking in its direction.
- a *mob*, when at least three birds were all on the same tree, closer to the stuffed owl, clearly interested in the stuffed owl and looking in its direction and alarming.
- the i*nitial number of birds at the feeder*, describing the number of birds that were at the feeder when the observer(s) arrived at the observation location.

If mobbing occurred, the observer(s) waited 5 min after the mob stopped to end the observation. If no mobbing occurred, the trial was stopped after 30 min. The end of mobbing was defined as when the birds went back to the feeder or left the area. Environmental information was also recorded during each trial such as the wind intensity (scale from 1 to 3), the temperature, the light conditions (sunny or cloudy) and the snow depth (using a ruler in the snow 5 cm away from the stuffed owl placement).

### 2.1 Statistical analyses

To assess the efficiency of the stuffed owl presence, we conducted a Pearson's Chi‐squared test with Yates' continuity correction comparing owl detection with and without the stuffed owl being present. For all the probability models, we used Generalized Linear Mixed Models using Template Model Builder (GLMMTMB), using the function ‘glmmTMB’ implemented in package ‘glmmTMB’ (Brooks et al., 2017) in R (R Core Team, 2021). Location ID was consistently included as a random factor and we considered morph (scored as a binary variable: G = grey, B = brown) as a factorial fixed effect. We furthermore included as fixed factors: the initial number of birds at the feeder present at the feeder, the season and the moment of the day (morning or afternoon). We included perturbation as a factorial variable scoring whether any perturbation (wind, rain, snow, people talking nearby, truck passing by the nearest road) occurred or not during the trial, because these could affect the behaviour of the passerines. The detection and mobbing probabilities were analysed as binomial GLMM scoring detection or mobbing as 1 and non‐detection or non‐mobbing as 0.

## 3 RESULTS

Antipredator responses (i.e. birds approaching the stuffed owl, birds mobbing) were recorded only when a stuffed owl was presented (experimental) and never when no stuffed owl was presented (control) (experimental 189/224, control 0/52; Chi‐square = 135.31, df = 1, *p* < .0001).

### 3.1 Mobbing probability in winter with and without snow cover

**FIGURE 3:**
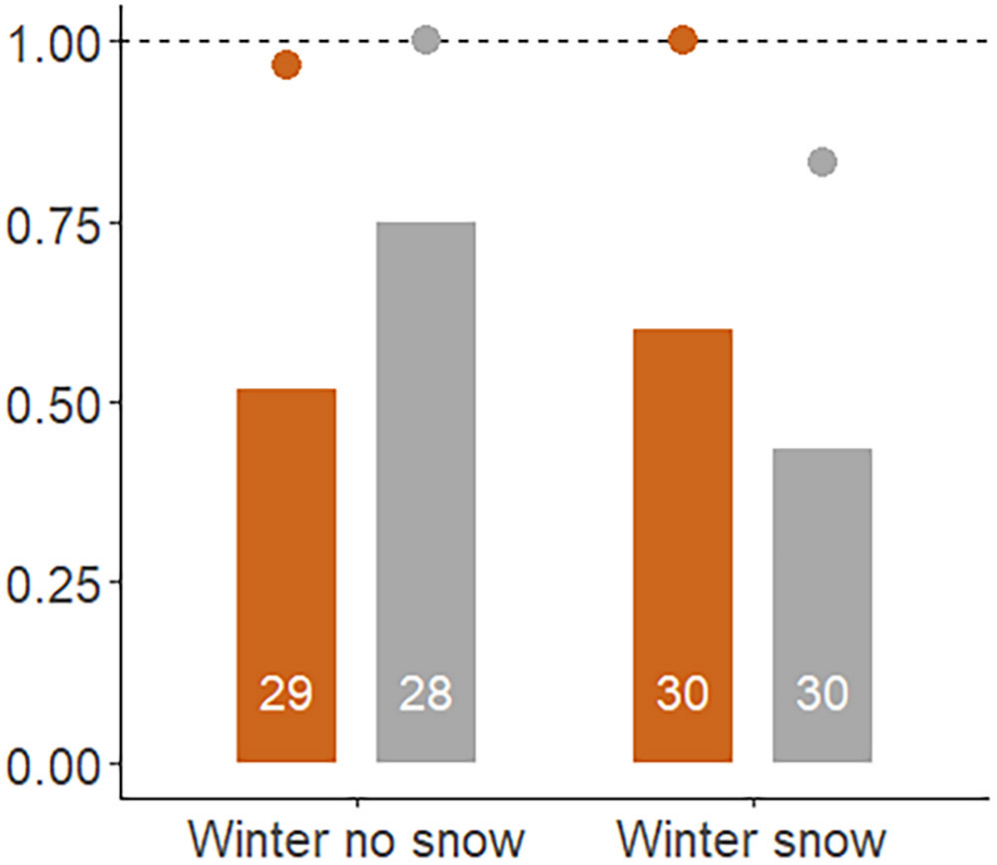
The proportion of all trials in which the stuffed owl was detected (dots) or mobbed (bars), when the stuffed owl was brown (plotted with brown colour) or grey (plotted in grey colour) and in winter without snow (‘no snow’) or in winter with snow (‘snow’). The dashed line indicates probability of 1. Plotted are the raw data, see Table 2 for analysis. The number of trials is indicated in the bars.

The probability for small passerines to detect the stuffed owl during the experimental trials was high in winter (dots in Figure 3); almost always the stuffed owl was detected. In approximately 60% of trials, the small passerines formed a mob, but the probability to mob the grey stuffed owl was higher than the probability to mob the brown stuffed owl in the absence of snow cover, but was the reverse when there was snow (significant interaction morph × snow cover in Table 2, bars in Figure 3).

**TABLE 2:** Analysis of deviance table (Type II Wald chi‐square tests) of a Generalized Linear Mixed Model using Template Model Builder analysing the mobbing probability of the stuffed owl by the mobbing birds in the winter season with or without snow cover (*n* = 117).

Variables	df	Chisq	*p*
*Mobbing probability—snow/no snow*			
Morph	1	0.02	.90
Season	1	1.91	.17
Perturbation	1	0.87	.35
Moment of the day	1	0.94	.33
Initial number of birds at the feeder	1	1.44	.23
Morph * Season	**1**	**4.35**	**<.05**

*Note*: The model includes the morph of the stuffed owl (grey/brown) and environmental information such as the season (Table 2: winter with snow, winter without snow; Table 3: spring, autumn), the initial number of birds present at the location, moment of the day (morning/afternoon) and perturbation (yes/no). The model includes an interaction between season and morph (annotated * in table). The location is considered a random factor. Significant fixed terms are indicated in bold font.

### 3.2 Mobbing probability in spring and autumn

**FIGURE 4:**
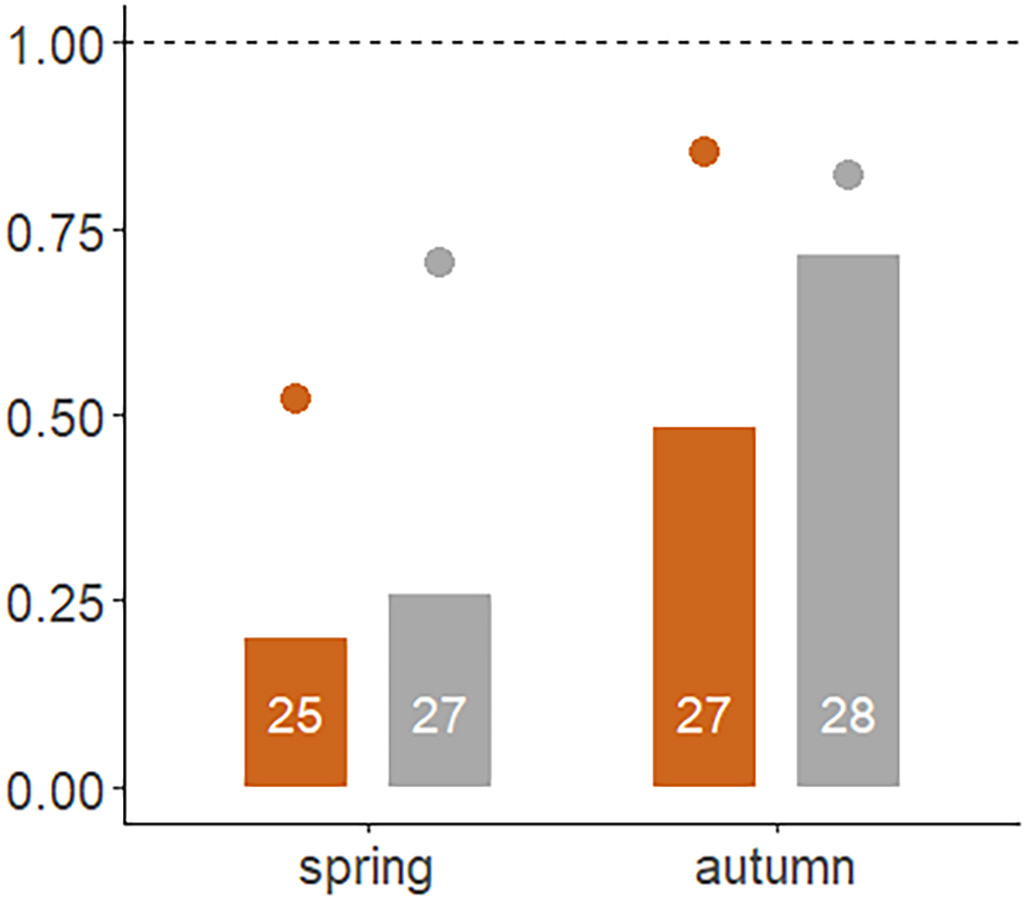
The proportion of all trials in which the stuffed owl was detected (dots) or mobbed (bars), when the stuffed owl was brown (plotted with brown colour) or grey (plotted in grey colour) and in the spring or autumn season. The dashed line indicates probability of 1. Plotted are the raw data, see Table 3 for analysis. The number of trials is indicated in the bars.

The probability for the owl stuffed owl to be detected in spring and autumn was about 80% and 60%, respectively (dots in Figure 4). The probability of being mobbed depended on the season and tended to be higher for the grey morph than the brown morph (bars in Figure 3; Table 3).

**TABLE 3:** Analysis of deviance table (Type II Wald chi‐square tests) of a Generalized Linear Mixed Model using Template Model Builder analysing the mobbing probability of the stuffed owl by the mobbing birds in spring and autumn (*n* = 107).

Variables	df	Chisq	*p*
*Mobbing probability spring/autumn*			
Morph	1	2.83	.09
**Season**	**1**	**7.55**	**<.05**
Perturbation	1	2.05	.15
Moment of the day	1	0.05	.83
Initial number of birds at the feeder	1	1.28	.26
Morph * Season	1	0.81	.37

*Note*: The model includes the same variables and random factors as the previous model (Table 3). Significant fixed terms are indicated in bold font. Interaction is annotated * in the table.

### 3.3 Detection probability all year around

The probability for the stuffed owl to be detected (dots in Figures 3 and 4) varied strongly across seasons (Analysis of deviance type II Wald Chi‐square test; Chisq = 10.97, df = 2, *p* < .01). However, detection did not differ between the two morphs, and the interaction between morph and season was not significant (Figures 3 and 4).

## 4 DISCUSSION

Our findings show that the lowered detection of the grey tawny owl morphs under snowy conditions as found earlier using pictures and human observers (Koskenpato et al., 2020) is likely to be operational under ecologically relevant conditions. Indeed, when investigating snow impact on mobbing probability, grey tawny owls have a significantly higher probability to be mobbed in winter without snow cover but have a significantly lower probability to be mobbed in winter with snow cover compared to brown tawny owls. Moreover, in the spring and autumn season, the brown morph enjoys a lower mobbing rate than the grey morph. Taken together, our experiments underline that the crypsis of the grey morph is highly context‐specific; the grey morph is only mobbed less by small passerines than the brown morph when there is snow.

Having a higher probability of being detected and mobbed by passerines during the daytime can be an issue for nocturnal predators. Indeed, nocturnal predators such as tawny owls rest during the daytime when diurnally active small passerines may find and mob them. When roosting in an exposed place, the tawny owl is detected and harassed and has to move to another spot (Hendrichsen et al., 2006). Moving entails using and hence losing energy that would otherwise be available, for example, for hunting the coming night. Our findings therefore imply that brown tawny owls may suffer costs relative to grey tawny owls, depending on whether there is snow or not during the energetically demanding winter season. On the other hand, the brown tawny owl morph appears well camouflaged in other seasons. More work is needed to elucidate whether the ecological process of detection and mobbing by small passerine prey in winter can create selective pressure on colour morph survival in the wild. Our findings would also benefit from replication or, ideally, experimental manipulation of snow cover as our comparison is made across different winters with and without snow. Hence, the presence/absence of snow cover thus is confounded with and uncontrolled for annual differences in our experiments. It is, in any case, noteworthy that in our study population, the survival of brown tawny owls is drastically lowered compared to the survival of grey tawny owls under snow‐rich conditions (Karell et al., 2011), which is consistent with the notion that snow‐rich winters are critical for survival.

We find that the detection and mobbing probability of tawny owls is particularly low in spring compared to autumn and winter. Our findings are similar to those reported by Dutour et al., 2017, showing higher mobbing intensity towards pygmy owls in autumn than in spring, which is possibly explained by seasonal variation in predator diet and predation pressure on passerines across seasons (Dutour et al., 2017). Heterospecific communication is also important in birds' choice to join a mobbing event and seasonal effects can occur in this context (Salis et al., 2023). Moreover, in spring, during the breeding season, passerines spend more time with conspecifics than during other times of the year when they form mixed‐species flocks (Ekman, 1989). During the breeding season, they might be more sensitive to conspecific mobbing calls, whereas at other times they may be sensitive to heterospecific mobbing calls (Dutour et al., 2019), since birds are often responding to other birds ‘by contagion’. Thus, the probability that passerines would react to another bird in the non‐breeding season is higher than that during the breeding season (Sieving et al., 2004). On the other hand, many studies have shown that the mobbing response to predators is higher during the breeding season (Altmann, 1956; Krams & Krama, 2002; Shedd, 1982, 1983). This could be related to temperature influencing mobbing responses in passerines like great tits (Cordonnier et al., 2023). It is also important to note that this study was conducted during only 1 year and thus the season effect could be confounded with a year effect.

Our results are in line with the observation that grey tawny owls survive better during severe winters with lots of snow and cold temperatures (Karell et al., 2011). Indeed, brown tawny owls have a higher probability to be mobbed during winter with snow cover, which might lead to energetic costs since they have to fly away to avoid mobbing. Moreover, the probability of detection is high in winter, independently of the morphs, which means that camouflage is particularly relevant during that season. Tawny owls might need to hide especially in winter to avoid predation risk and mobbing. We have previously showed in a behavioural experimental setup that brown tawny owls were more likely to use exposed perches than grey tawny owls after release in a novel environment (Perrault et al., 2023). Thus, the two morphs might display different strategies in order to avoid predation risk and mobbing. Nevertheless, several other factors may affect winter survival; for example, brown tawny owls have poorer insulation of their back feathers than the grey morph (Koskenpato et al., 2016).

Our findings demonstrate that the light (grey) tawny owl is critically dependent on snowy winters for camouflage during daytime roosting. Under global warming, winters in northern latitudes will become less snowy. As a consequence, the grey tawny owl morph may lose its camouflage advantages. In general, for colour‐polymorphic species with morphs that are adapted to specific environmental conditions, climate change challenges their ability to camouflage and the advantages they might have in specific environments. Pronounced changes, possibly including strong declines in population, can hit colour‐polymorphic species adapted to cold environments (Mills et al., 2018; Zimova et al., 2016).

## AUTHOR CONTRIBUTIONS

**Charlotte Perrault:** Data curation (lead); formal analysis (lead); funding acquisition (equal); investigation (lead); methodology (equal); visualization (lead); writing – original draft (lead); writing – review and editing (lead). **Chiara Morosinotto:** Conceptualization (equal); formal analysis (equal); investigation (equal); methodology (equal); supervision (equal); validation (equal); writing – original draft (equal); writing – review and editing (equal). **Jon E. Brommer:** Formal analysis (equal); funding acquisition (equal); investigation (equal); supervision (equal); validation (equal); writing – original draft (equal); writing – review and editing (equal). **Patrik Karell:** Conceptualization (equal); formal analysis (equal); funding acquisition (equal); investigation (equal); project administration (equal); supervision (equal); validation (equal); writing – original draft (equal); writing – review and editing (equal).

## FUNDING INFORMATION

The research was funded by Societas pro Fauna et Flora Fennica, the EDUFI fellowship and the Biology, Geography and Geology (BGG) graduate school at the University of Turku (to CP) and the Academy of Finland (projects 309992, 314108, and 335335, to PK; 321471 to JEB). CM acknowledge for the support of NBFC to University of Padova (funded by the Italian Ministry of University and Research, PNRR, Missione 4 Componente 2, ‘Dalla ricerca all'impresa’, Investimento 1.4, Project CN00000033).

### OPEN RESEARCH BADGES

This article has earned an Open Data badge for making publicly available the digitally‐shareable data necessary to reproduce the reported results. The data is available at https://data.mendeley.com/datasets/8bwmxk2ddb/1 (doi: 10.17632/8bwmxk2ddb.1).

## Supporting information


Data S1.

## Data Availability

The datasets generated during and/or analysed during the current study are available in the Mendeley data repository, web link for dataset https://data.mendeley.com/datasets/8bwmxk2ddb/1.
